# New Approaches to the Creation of Highly Efficient Pd-Ag and Pd-Cu Membranes and Modeling of Their Hydrogen Permeability

**DOI:** 10.3390/ijms252312564

**Published:** 2024-11-22

**Authors:** Iliya Petriev, Polina Pushankina, Michail Drobotenko

**Affiliations:** 1 Research Institute of Hydrogen Energy, Kuban State University, Krasnodar 350040, Russia; 2 Laboratory of Problems of Stable Isotope Spreading in Living Systems, Southern Scientific Centre of the RAS, Rostov-on-Don 344006, Russia

**Keywords:** palladium-containing membranes, surface activation, nanoparticles, nanowires, hydrogen permeability, steam reforming of hydrocarbons

## Abstract

Thin-film membranes of Pd-Ag and Pd-Cu alloys capable of releasing hydrogen in a wide temperature range have been developed. The surface activation of the membranes with a nanostructured coating made it possible to intensify hydrogen transport through Pd-containing membranes at low temperatures. This effect was achieved by accelerating limiting surface processes by increasing the active area of the membrane. Surface-activated membranes demonstrated the highest values of hydrogen flux over the entire temperature range, which reached up to 49.4 mmol s^−1^ m^−2^ for Pd-Ag membranes and up to 32.9 mmol s^−1^ m^−2^ for Pd-Cu membranes. Membranes modified with filiform nanoparticles demonstrated a hydrogen flux up to 12 times higher than that of membranes with a smooth surface. Based on the results obtained, a theoretical model of hydrogen transport through metal membranes was developed, taking into account the effect of the state of the membrane surface on hydrogen transport at low temperatures. This model makes it possible to predict hydrogen flows in the entire temperature range much more accurately compared to other existing models. The selectivity and stability of the developed membranes over a long period of operation have been confirmed. The study of the effect of the surface activation of Pd-based membranes on the intensification of hydrogen permeability has shown the success of the method developed, which in turn opens up wide opportunities for creating low-temperature, highly efficient membrane hydrogen filters based on palladium and other devices based on them.

## 1. Introduction

Hydrogen has promising prospects as an energy carrier [1,2,3,4,5]. This is due to its high energy density per unit mass and the possibility of obtaining it from a wide range of raw materials [6,7,8]. Such raw materials include hydrocarbons, including fossil fuels [9], waste [10], and biomass [11], as well as ammonia [12] and water [13]. Despite the development of new techniques and methods for obtaining hydrogen from renewable sources, today, the majority of industrial hydrogen (about 80%) is produced by steam reforming from natural gas [14]. This process often uses traditional reactors that require additional stages of purification of the product—hydrogen—to a certain purity required for further use (for example, fuel cells require hydrogen with a purity of 99.99% [15,16]) [17,18,19]. Thus, the inclusion of additional purification devices in reforming can increase the energy consumption of such a plant by up to 50%, which significantly affects the final cost of the hydrogen obtained [20]. A promising alternative is to replace traditional reactors with membrane reactors, which are capable of isolating high-purity hydrogen from a gas mixture in a single reaction zone, with a significant reduction in energy costs [21,22]. The advantages of membrane reactors in terms of design are ease of operation and maintenance and the possibility of scaling for large and decentralized enterprises [23,24,25]. The basis of such reactors are hydrogen-permeable membranes, which have a unique set of properties that can ensure efficient operation [26].

Today, the most popular membrane materials are those based on palladium, which have virtually infinite selectivity and high hydrogen permeability [27,28,29,30]. The introduction of such membranes into the reactor design provides a shift in the reaction, allowing the processes of hydrogen extraction and purification to be combined in one device (reactor) [6,31,32,33]. However, such membranes have a number of disadvantages, including the embrittlement of pure palladium in a hydrogen atmosphere during thermal cycling, rapid surface deactivation, effective operation only at high temperatures, and high material cost [34,35,36]. Considerable efforts are focused on creating membranes with high mechanical and chemical resistance capable of ensuring the effective operation of a hydrocarbon steam reforming reactor.

The solution to the problem of embrittlement of the membrane material—palladium—is its alloying with other metals, which include Ag, Cu, Au, Ni, and Ru [37,38,39]. Thus, S. Pati et al., in their work [40], investigated the permeability of membranes made of Pd_57_Cu_43_ alloy for hydrogen. It was found that membranes of Pd-Cu alloy have stable characteristics in the presence of C_3_H_6_ compared with metal membranes of Pd and demonstrate high conversion and improved hydrogen extraction. M. Omidifar and A.A. Babaluo, in their work, reported [41] a new strategy for the preparation of metal (Pd-Ni)/ceramic membranes, which can be used as an alternative to conventional expensive Pd membranes. The Pd-Ni composite membrane showed ideal permeability stability for 145 h at 450 °C and infinite selectivity for H_2_/N_2_. Z. Yin et al., [42] in their work, investigated hydrogen flux through Pd-Ru membranes after exposure to H_2_S in various concentrations and for various periods of time. According to the results obtained, the presence of Ru on the surface significantly prevents Pd sulfidation, and, therefore, Pd-Ru membranes demonstrate excellent tolerance to sulfur. The most promising membrane systems are recognized as palladium–silver and palladium–copper alloys [26,28,43]. Such combinations effectively prevent the hydrogen embrittlement of the material at low temperatures, have high mechanical and thermal stability, excellent permeability and selectivity for hydrogen, and relatively low costs due to a significant decrease in the % palladium content [44,45,46]. However, this approach does not fully reduce the problems of rapid deactivation of the membrane surface caused by fouling and/or poisoning and low permeability at low temperatures [47,48].

The listed problems can be partially solved by membrane surface activation. One such method is the creation of catalytically active ‘cracks’ by alternating electrolytic oxidation or reduction and prolonged exposure to a glow discharge or calcination in air [49]. Thus, to study the positive effects of such membrane activation on hydrogen transfer, N. Vicinanza et al. [50] subjected Pd77%Ag23% membranes to a three-stage heat treatment in air. The study revealed that heat treatment in air increased the effective surface area of the membrane, which affected the increase in hydrogen permeability after each stage of treatment. However, this activation method in practice turns out to be ineffective for solving the problems presented. A new approach is membrane surface modification, which allows for a significant improvement in the properties and characteristics of the material for many areas of its application [51,52,53]. In particular, the use of metal nanoparticles as modifiers shows significant promise [54,55,56,57,58]. Thus, the application of a developed nanostructured coating to the surface of a Pd-containing hydrogen-permeable membrane can significantly increase its stability and permeability at low temperatures [59,60].

The operation of palladium-based membranes at low temperatures is poorly understood, and existing theoretical concepts are unable to fully describe the processes occurring. In this paper, for the first time, the main attention is paid to improving the understanding of the process of hydrogen transfer by Pd-based membranes under low-temperature conditions.

## 2. Results and Discussion

### Study of Hydrogen Transfer Through the Manufactured Surface-Activated Pd-Ag and Pd-Cu Membranes

**Figure 1:**
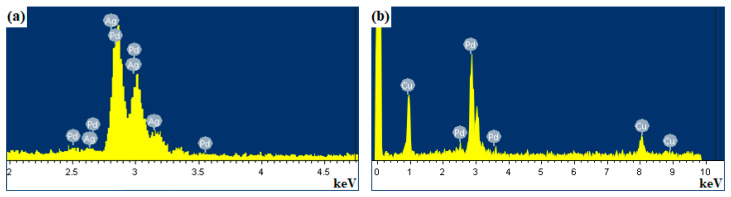
EDS spectra of the elemental composition of Pd-23%Ag (**a**) and Pd-40%Cu (**b**) alloy films.

During the study, samples of Pd-23%Ag and Pd-40%Cu alloy films were manufactured. The addition of a less noble metal to the base alloy of the hydrogen-permeable membrane—Pd-Ag and Pd-Cu films—was necessary to improve the mechanical properties of the material, especially under low operating temperatures. The main degradation mechanism is hydrogen embrittlement occurring mainly during thermal cycling in a hydrogen atmosphere and the saturation of the metal lattice with hydrogen. Numerous transitions from the α to the β phase and back lead to deformation and disrupt the integrity of the metal lattice. Thus, a change in the geometry of the palladium lattice upon hydrogen absorption leads to hydrogen-phase hardening [61], caused by the occurrence of internal plastic deformation and the development of specific processes of interaction of dissolved hydrogen, hydrogen-containing phases, and hydrogenated defects of the crystal structure. As a result of hardening, an increase in the hydrogen content in the region of coexistence of both the α- and β-phases leads to a change in their substructure. Hydrogen-phase cold working has a significant effect on the distribution pattern and internal structure of β-phase crystals in the α-phase matrix and is one of the causes of hysteresis observed during hydrogen adsorption and desorption in the (α ↔ β) transition region [62]. Therefore, to solve such problems, alloying was performed in this work, and palladium alloys with Cu and Ag were prepared. Palladium alloys with metals with an isomorphic lattice (Ni, Co, Fe, Ag, Au) crystallize with the formation of continuous solid solutions. Alloying elements with a hexagonal structure strengthen palladium more strongly but also reduce its ductility more strongly than metals with a cubic structure. The doping of palladium affects hydrogen diffusion inside the membrane, the rate of dissolution and release of hydrogen atoms, the recombination and dissociation of molecules, and, to a lesser extent, adsorption and desorption. As a result of palladium doping, the temperature of the hydride phase transition, the resistance to dilation, and the hydrogen permeability coefficient change in relation to those of pure palladium. Alloying also allows for a reduction in the precious metal content in the resulting alloys, which provides a significant (up to three-fold) reduction in the cost of the final product. Such a reduction in cost is also explained by the fact that the specific density of copper is lower than that of Pd, which in the same mass ratio provides additional savings of precious metal for the same volume of material. The composition of the films obtained was controlled by energy-dispersive X-ray spectroscopy (EDS) (Figure 1). EDS analysis demonstrated a uniform distribution of elements in the films. Thus, according to the results obtained, the content of elements in the Pd-Ag film was 76.93% palladium and 23.07% silver. The content of elements in the Pd-Cu film was 60.42% palladium and 39.58% copper.

**Figure 2:**
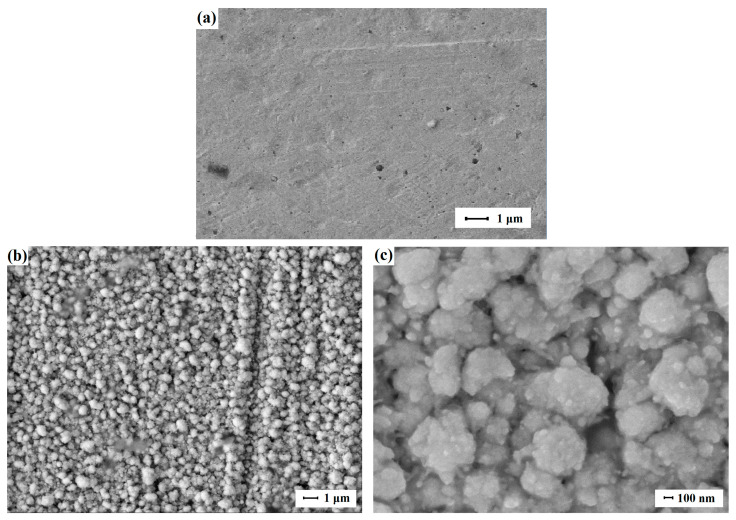
(**a**) SEM image of the surface of the unmodified Pd-Cu film. (**b**,**c**) SEM images of the modifying nanostructured palladium coating synthesized by the classical electrodeposition technique from a H_2_PdCl_4_ solution at a current density of 5–6 mA cm^−2^ and a time of 1.5–3 min.

The surface of the prepared smooth Pd-Ag and Pd-Cu films was activated by applying a modifying nanostructured palladium coating to intensify the hydrogen flux density at low temperatures (<200 °C). The first series of samples was modified using the classical technique of electrolytic deposition from a palladium chloride solution. The morphology of the obtained nanostructured palladium coatings was studied by scanning electron microscopy (SEM). Figure 2 (Pd-Cu) and Figure S1 (Pd-Ag) show SEM images of smooth film samples without modifier and low- and high-magnification SEM images of the modifying nanostructured palladium coating synthesized by the classical method. Minor differences in the morphology of nanostructured coatings deposited on substrates made of different materials are due to the choice of the SEM study area. However, extensive analysis confirms the most similar morphology and particle size in the studied samples. The resulting Pd particles had a spherical shape with an average size of about 90–110 nm and also formed larger micrometer agglomerates.

**Figure 3:**
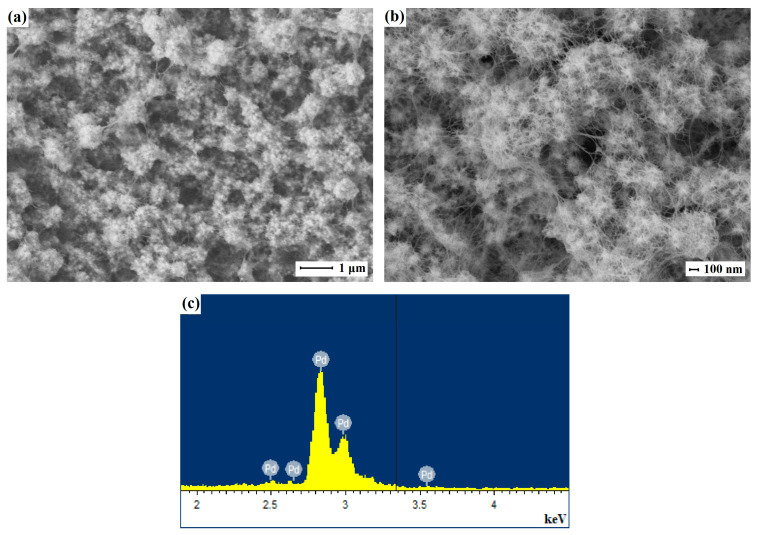
SEM images of the modifying nanostructured palladium coating based on nanothreads synthesized by two-step electrodeposition from a solution of H_2_PdCl_4_ with C_19_H_42_BrN at a current density of 2.5 μA cm^−2^ for 30–40 s and a current density of 0.35 mA cm^−2^ for 3–5 min (**a**,**b**) and EDS analysis (**c**).

The second series of samples of the manufactured smooth Pd-23%Ag and Pd-40%Cu films was modified using the authors’ technique with a nanostructured palladium coating based on nanoparticles of non-standard morphology. The resulting coating consisted of elongated palladium particles shaped like threads, with a cross-sectional diameter lying in the range of 5–10 nm. Such nanoparticles, unlike classical spherical ones, grow in one direction and, due to this, have an elongated shape. The specific properties of such particles depend on the geometric anisotropy factor—the ratio of length to diameter. During synthesis, a surfactant and deposition current were used as tools for tuning and controlling the morphology of the nanostructured coating [59,63]. A correctly selected concentration of the surfactant prevented the particles from rounding during growth and directed the growth of the particles in a given direction. A two-step change in current density facilitated the nucleation process required for self-assembly into larger particles with the desired properties. Subsequent increases in current allowed for directional particle growth while maintaining the seed geometry. Figure 3 (Pd-Cu) and Figure S2 (Pd-Ag) show SEM and EDS images of the nanothread-based modified palladium nanostructured coating.

The manufactured film samples were modified with two different types of coatings to compare the effect of different morphologies of surface activators on gas transport characteristics.

**Figure 4:**
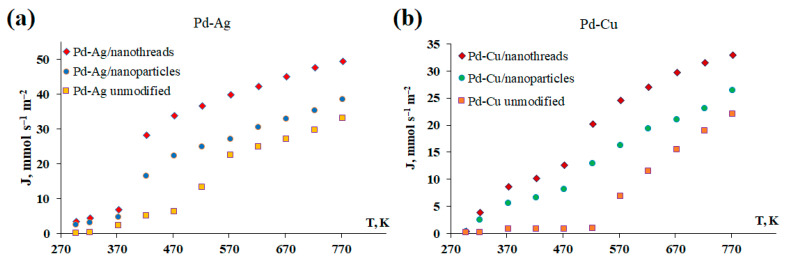
Temperature dependence of hydrogen flux through the developed Pd-Ag (**a**) and Pd-Cu (**b**) membranes.

The manufactured surface-activated films of Pd-23%Ag and Pd-40%Cu alloys were studied as membranes in hydrogen transport processes. A series of experiments was conducted to study the effect of morphology on hydrogen transport by the developed membranes. In the first series of experiments, the hydrogen flux was studied in a wide temperature range from room temperature (298 K) to 773 K. This range was chosen to evaluate the temperature corridor of the efficiency of surface-activated membranes. Figure 4 shows the graphs of the temperature dependence of the penetrating hydrogen flux through the developed Pd-Ag and Pd-Cu membranes. According to the results, the surface-activated membranes demonstrated an increase in flux in almost the entire temperature range. Pd-Ag and Pd-Cu membranes modified with nanowires demonstrated a hydrogen flux of up to 49.4 mmol s^−1^ m^−2^ and 32.9 mmol s^−1^ m^−2^, respectively, while the fluxes for membranes activated with classical nanoparticles were slightly lower at up to 38.4 mmol s^−1^ m^−2^ and 26.3 mmol s^−1^ m^−2^, respectively. The fluxes obtained were up to 1.5 times higher than those of membranes with a smooth surface. The greatest difference in fluxes was especially noticeable in the temperature range from 298 to 473 K, where, according to the classical theory, surface processes prevail or completely limit hydrogen transfer. Thus, surface-activated membranes demonstrated fluxes up to 12 times higher in this temperature range than membranes without a modifier. The results obtained allow us to speak about the effective influence of surface activation on the intensification of hydrogen flux through Pd-based membranes.

**Figure 5:**
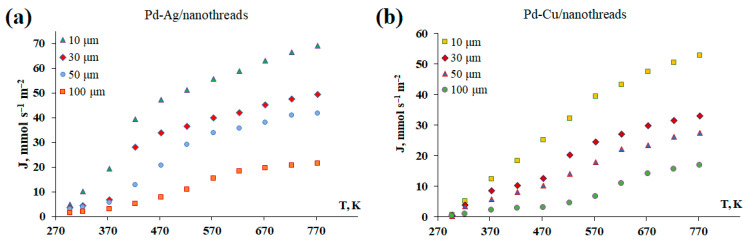
Temperature dependence of hydrogen flux through the developed Pd-Ag (**a**) and Pd-Cu (**b**) membranes of different thicknesses.

The dependence of the permeability of Pd-Ag and Pd-Cu membranes modified with nanothreads on their thickness was investigated. Manufactured surface-activated Pd-Ag and Pd-Cu membranes of different thicknesses from 10 to 100 μm were studied in hydrogen transfer processes in a temperature range from 298 to 773 K. In the low-temperature range, membrane activation allowed for the acceleration of surface processes, thereby reducing the effect of the limiting stage at low temperatures. As a result, a transition to limitation by processes occurring in the membrane volume, i.e., diffusion, was observed. The obtained data, presented in Figure 5, demonstrate the inverse dependence of the hydrogen flux on an increase in the thickness of the Pd-based membrane. The increase in the hydrogen flux through a membrane 10 μm thick was increased up to three-fold at 773 K compared to that through a membrane 100 μm thick. The samples were tested for 50 h and demonstrated high mechanical stability, which was confirmed by purging with inert gas—helium—and chromatographic testing. A membrane with a thickness of 30 μm was selected for the tests as having the most optimal strength and permeability characteristics.

To date, there are quite a few works in the global literature on low-temperature hydrogen permeability through membranes based on Pd. The existing works differ greatly in the data obtained and, for the most part, do not include studies in temperature ranges below 100 °C. Table 1 provides a comparative analysis of the results obtained in this study with data from literary sources. The developed membranes had fairly high permeability indices, not inferior to those of most analogs.

**Figure 6:**
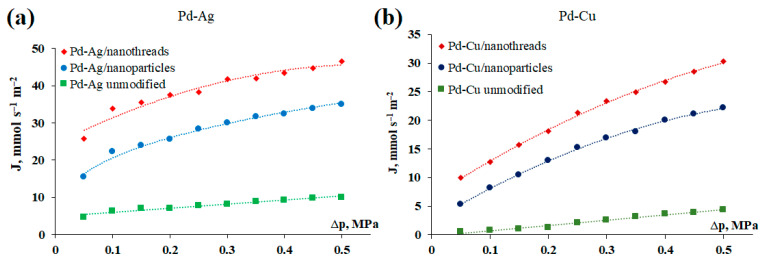
Dependence of hydrogen flux on excess pressure for the developed Pd-Ag (**a**) and Pd-Cu (**b**) membranes.

In the second series of experiments, the dependence of the penetrating hydrogen flux on the pressure difference in the range of 0.05–0.5 MPa was investigated. The measurements were carried out at a temperature of 473 K, since at this temperature, the greatest difference in the magnitude of hydrogen flows between surface-activated and smooth membranes was recorded. Figure 4 shows the graphs of the dependence of the hydrogen flux on excess pressure for the developed Pd-Ag and Pd-Cu films. The results obtained showed a similar picture to the previous experiment. The Pd-Ag and Pd-Cu membranes modified with nanowires demonstrated the highest hydrogen flux of up to 46.5 mmol s^−1^ m^−2^ and 30.3 mmol s^−1^ m^−2^, respectively. The obtained values were 1.5 times higher than those for the Pd-Ag and Pd-Cu membranes activated with classical nanoparticles, at up to 34.9 mmol s^−1^ m^−2^ and 22.1 mmol s^−1^ m^−2^, respectively. Surface-activated membranes demonstrated an increase in values of up to seven times those of non-activated membranes. This experiment also allows us to graphically visualize the influence of one or another limiting stage (surface processes and/or diffusion) on hydrogen transfer through the developed membranes. The hydrogen flux passing through the membrane can be described by the following equation [71]:

(1) $$J_{H_{2}}=\frac{P_{H_{2}}}{\delta }(p_{1}^{n}-p_{2}^{n})$$

where $J_{H_{2}}$ is the hydrogen permeation flux; $P_{H_{2}}$ is the hydrogen permeability; *δ* is the membrane thickness; $p_{1}^{n}\;$ and $p_{2}^{n}$ are the partial pressure on the inlet and outlet sides of the membrane, respectively; and *n* is the pressure exponent. The value of the pressure exponent *n* lies in the range from 0.5 to 1. At the boundary positions at *n* = 0.5, the hydrogen transport process is completely limited by diffusion, while at *n* = 1, the hydrogen transport is limited by surface processes (dissociative adsorption and/or recombinant desorption) [72]. According to the experimental results, the data presented in Figure 6 for both non-activated Pd-Ag and Pd-Cu membranes with a smooth surface are well approximated by a first-order line and have an exponent *n* equal to 1, which may indicate that hydrogen transport is limited by surface processes at the selected temperature. The result obtained fully corresponds to the classical ideas about the limitations of hydrogen transfer by surface stages at temperatures below 473 K. For activated membranes, a different picture was observed; the exponent *n* was in a range from 0.75 to 0.89. Such a result may indicate a decrease in the influence of surface processes and a transition to the limitation of hydrogen transport by a combination of the diffusion stage in the membrane volume and dissociative–associative stages. This phenomenon can be explained by an increase in the adsorption activity of the membrane surface due to an increase in the number of vacant active centers. Presumably, this is the reason for the decrease in the energy barrier of dissociation and/or recombination of hydrogen molecules on the membrane surface, which leads to an increase in the rate of hydrogen penetration.

**Figure 7:**
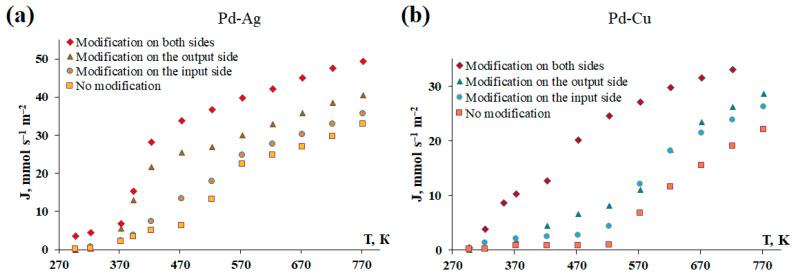
Temperature dependence of the hydrogen flux through Pd-Ag (**a**) and Pd-Cu (**b**) membranes surface-activated on both sides, on the input/output side, and with a smooth surface.

In connection with the obtained results, the essence of the third series of experiments was to determine the contribution of each individual stage (adsorption, desorption, diffusion) to the process of hydrogen transfer through the developed membranes. For this purpose, membranes surface-activated from the input/output side, both sides, and with a smooth surface were studied. The experiment was carried out on two types of Pd-Ag and Pd-Cu membranes to confirm the repeatability of the experimental results. Figure 7 shows the results of the temperature dependence of the hydrogen flux passing through the Pd-Ag and Pd-Cu membranes. Non-activated membranes with a smooth surface demonstrate fairly low values of hydrogen flux up to 473 K, after which they begin to steadily increase. The result observed is completely consistent with the classical concepts of hydrogen transfer by membranes based on Pd: at a temperature of about 473 K, a transition from the surface-limited mode to the diffusion-limited mode occurs. However, the picture observed for membranes whose surface has been activated by applying a nanostructured coating is quite different. Membranes activated only from the input side demonstrate an increase in hydrogen flux as early as 423 K. This can be explained by the acceleration of the adsorption stage in the process of hydrogen transfer. For membranes activated only from the output side, a rapid increase in the flux is observed as early as 373 K. This result could be due to the acceleration of the desorption stage, which, as can be seen, has a prevailing effect compared to adsorption. Membranes activated from both sides demonstrated a continuous rapid increase in flux over the entire temperature range. Probably, this result was achieved due to the acceleration of both surface stages. In accordance with the results obtained, we can discuss a shift in the limitation of the hydrogen transfer process by diffusion toward lower temperatures for membranes activated from one side. For membranes with a nanostructured coating applied to both sides, the transition to diffusion limitation occurs almost immediately or is weakly recorded at the initial low temperatures. Thus, surface-activated membranes demonstrate flows several times higher than membranes with a smooth surface. The greatest difference in the fluxes was recorded in the low-temperature range (298–473 K) and reached more than an order of magnitude. Surface-activated membranes retained a high hydrogen flux over the entire studied temperature range (298–773 K), which was higher than that of membranes with a smooth surface even at high temperatures. The results obtained allow us to conclude that the application of a nanostructured modifying coating can significantly intensify the hydrogen flux through Pd-containing membranes at low temperatures, when surface processes are limiting.

However, the results of the study of surface-activated membranes demonstrated deviations from the generally accepted classical model. The classical model describes the phenomenon of hydrogen transfer through smooth membranes with a single surface roughness. In view of this, the model may be valid for the high-temperature operating mode, in which the desorption rate makes an insignificant contribution to hydrogen transport through the membranes. Previously, modeling low-temperature processes of hydrogen transfer through Pd membranes was extremely challenging due to the difficulties in recording and the greatly varying data in the few, virtually non-existent studies. However, the membrane modification described in this work made it possible to study the previously hard-to-reach range of low temperatures and to identify clear patterns. The results of this experimental study indicate the presence of multiple surface-active vacancies for hydrogen atoms in the developed surface-activated membranes, which complicates the modeling of transition processes from the surface to the bulk and back. In view of this assumption, the solution of the model in the low-temperature range yields a theoretical result below the experimental one due to the underestimated value of the limiting surface processes. It follows that the classical model requires improvement; therefore, the membrane surface roughness coefficient was introduced.

**Figure 8:**
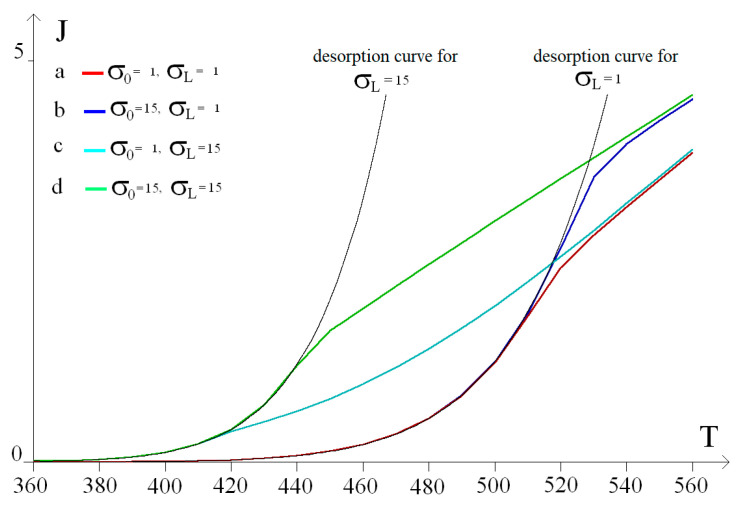
Model graphs of the temperature dependence of the hydrogen flux passing through membranes with different surface roughness.

The calculation results are shown in Figure 8 as graphs of the hydrogen flux J versus temperature T and roughness of the input and output surfaces. Graph (a) corresponds to an unmodified smooth membrane (*σ*_0_ = 1, *σ_L_* = 1), graph (b) to a membrane with a modified output surface (*σ*_0_ = 1, *σ_L_* = 15), (c) to a flow graph for a membrane with a modified input surface (*σ*_0_ = 15, *σ_L_* = 1), and (d) to a membrane with modified input and output surfaces (*σ*_0_ = 15, *σ_L_* = 15). The graphs of the desorption flux on the output surface at its full saturation (*θ_L_* = 1) for different values of output surface roughness (*σ_L_* = 1 and *σ_L_* = 15) are shown in black.

It is evident from Figure 8 that the flow graphs at low temperatures are limited by desorption curves; with increasing temperature, diffusion becomes the limiting factor. Thus, desorption on the outlet surface, which limits hydrogen transport at low temperatures, is determined by the membrane surface roughness; therefore, the flow graphs with the same *σ_L_* value are similar at low *T* values. In addition, from the calculated graphs as well as from the experimental ones, one can see the effect of increasing the input surface roughness on the value of the passing flow (*J_ads_*–*J_des_*). An increase in the roughness *σ*_0_ leads to an increase in the degree of saturation of the membrane input surface *θ*_0_ and, consequently, to an increase in the near-surface hydrogen concentration *C*_0_ in the Pd array. With a gradual transition to diffusion limitation, the values of the hydrogen flows passing through membranes with different surface roughness values reach a common plateau, which is due to a decrease in the influence of surface processes with increasing temperature.

Thus, the experimental results of hydrogen transfer through the developed surface-activated membranes qualitatively coincide with the theoretical ones, which allows us to speak about the practical applicability of the improved model.

**Figure 9:**
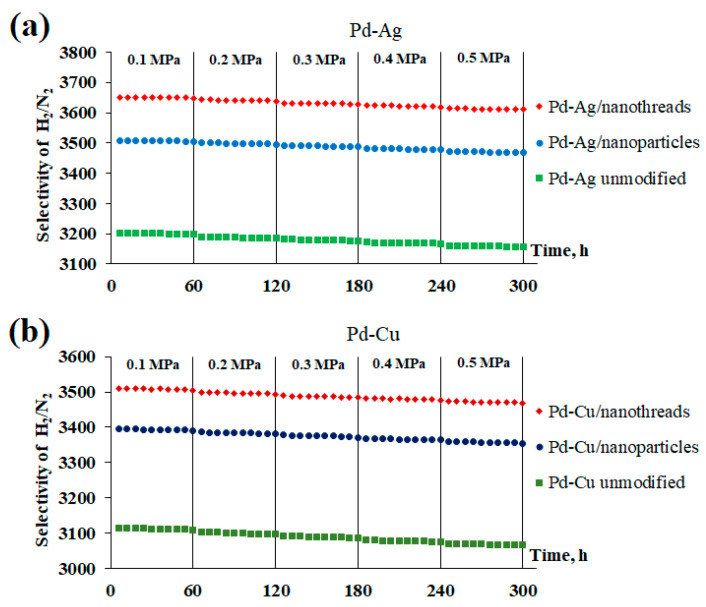
Dependence of selectivity on excess pressure through the developed Pd-Ag (**a**) and Pd-Cu (**b**) membranes.

In addition to permeability, an important characteristic of membranes is their hydrogen selectivity. In this series of experiments, the selectivity of the developed Pd-Ag and Pd-Cu membranes was studied by testing hydrogen permeation and nitrogen leakage at 473 K in a pressure range from 0.1 to 0.5 MPa. Figure 9 shows data from long-term H_2_/N_2_ permeation studies for two surface-activated types of coatings and smooth Pd-Ag and Pd-Cu membranes, recorded for 300 h. Hydrogen transfer through metal membranes follows the dissolution–diffusion mechanism, while nitrogen leakage through the membrane occurs through defects. Therefore, an increase in the transmembrane pressure difference can lead to an increase in the nitrogen flux. During the experiment, a very insignificant decrease in selectivity was observed in the pressure range under study. According to the results, all the developed membranes demonstrated a high degree of selectivity throughout the entire experiment. The highest values of H_2_/N_2_ selectivity at a pressure of 0.5 MPa were observed for membranes surface activated with filiform nanoparticles, reaching up to 3611 for the Pd-Ag membrane and up to 3469 for the Pd-Cu membrane. The obtained selectivity values for surface-activated membranes were up to 1.2 times higher than those for membranes with a smooth surface. It should be noted that the hydrogen flow was stabilized each time at a fixed pressure value, and the nitrogen leakage did not increase either. Thus, the manufactured membranes demonstrated stability and resistance to pressure drops over a long period of time, as well as the absence of significant mechanical defects in the form of holes and seals.

## 3. Materials and Methods

### 3.1. Fabrication of Pd Membranes: Creation of an All-Metal (Self-Supporting) Base Based on Pd Alloys and Its Modification

The membranes based on binary alloys (Pd-Ag and Pd-Cu) studied in the work were fabricated by melting the alloy components in an electric arc furnace. The metal ingots were immersed in a copper crucible and melted in a chamber at an inverter current of 20 to 120 A for Pd-Ag and 30 to 90 A for Pd-Cu. The resulting ingots of Pd-23%Ag and Pd-40%Cu alloys were rolled with intermediate annealing on DRM–130 rollers (Durston, High Wycombe, UK) to the required film thickness of 30 μm.

The elemental composition of the films was controlled by energy-dispersive X–ray spectroscopy on an INCA semiconductor energy-dispersive attachment (Oxford, UK) as part of a JSM–7500F scanning electron microscope (JEOL, Tokyo, Japan).

The surface of the films was activated by applying a nanostructured modifier using electrolytic deposition on a P–40X potentiostat–galvanostat (Elins, Moscow, Russia).

The first series of Pd-Ag and Pd-Cu film samples was modified using the classical technique. The binary alloy films were pre-cleaned and degreased in ethanol and NaOH solution, respectively. The film samples were placed in a working two-electrode cell with a palladium counter electrode and polarized at a current density of 10–20 mA cm^−2^ anodically in 0.1 M HCl and cathodically in 0.05 M H_2_SO_4_. The cell with the prepared samples was then filled with a working solution of 2% H_2_PdCl_4_. The nanostructured Pd coating was deposited at a current density of 5–6 mA cm^−2^ for 1.5–3 min. Upon the completion of the process, the samples obtained were washed with double-distilled water.

The second series of samples was modified in accordance with the authors’ technique. The samples of Pd-Ag and Pd-Cu films were prepared according to the technique described for the first series. The cell with the prepared samples was filled with a working solution containing 2% H_2_PdCl_4_ with C_19_H_42_BrN. The main difference of this technique was the two-stage deposition of the nanostructured Pd coating: the growth of nanoparticles was initiated by applying a current of 2.5 μA cm^−2^ for 30–40 s to start the slow growth of nuclei and ensure the adaptation of the system to the applied current. The current was then increased to 0.35 mA cm^−2^ for 3–5 min to continue the growth at a rate fast enough to obtain the desired particle morphology while maintaining the seed geometry. Upon the completion of the process, the samples obtained were washed with double-distilled water.

The surface morphology of the modified membranes was studied using a JSM–7500F scanning electron microscope (JEOL, Japan). The processing of microscopy data and the calculation of statistical parameters were performed using the Gwyddion-2.55 data visualization and analysis program.

### 3.2. Study of Gas Transport Characteristics of the Manufactured Membranes

**Figure 10:**
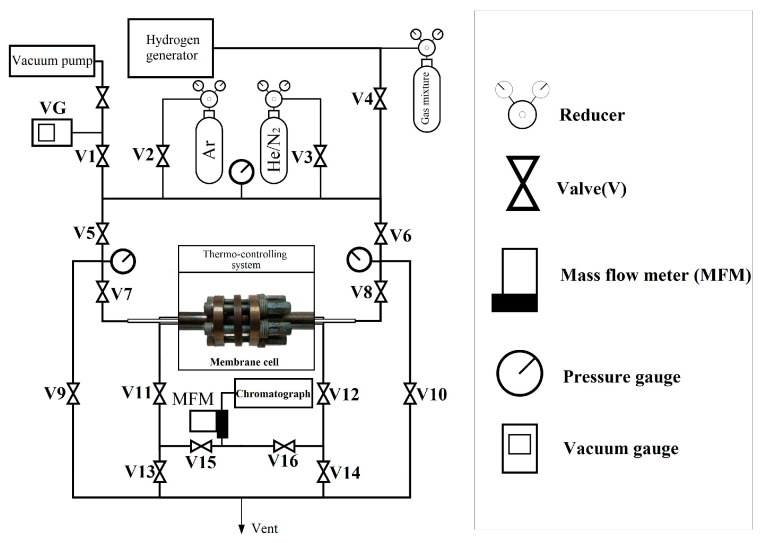
Schematic representation of the setup for measuring H_2_ permeability.

The gas transport characteristics of the manufactured Pd-Ag and Pd-Cu membranes were studied using a hydrogen permeability measuring device. The Pd-Ag and Pd-Cu alloy membrane samples were mounted in a working cell that ensured reliable fixation and sealing of the sample and were fixed using a flange joint. Before the main H_2_ permeability tests, the cell was purged with an inert gas, He, in order to detect sample defects and leaks. The system was evacuated, and the H_2_ permeability experiments of the membrane samples were carried out in a temperature range of 298–773 K and at pressures of 0.05–0.5 MPa. The gas supply to the system was controlled using a gas mass flow controller that provided the required flow. The vacuum level in the system was controlled using a vacuum gauge. The membrane selectivity was studied by the H_2_/N_2_ flow ratio. The setup diagram is shown in Figure 10.

### 3.3. Model of Hydrogen Transfer Through a Surface-Activated Membrane

The mathematical model used in this work was based on the model created by T.L. Ward and T. Dao and described in [73].

**Figure 11:**
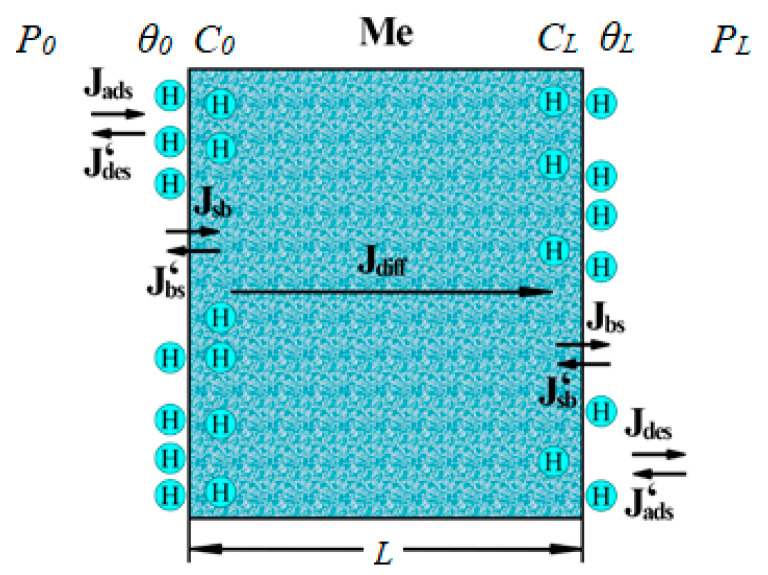
Schematic representation of the stages of hydrogen transfer through a Pd-containing membrane.

Description of the problem and notations:

The mechanism of hydrogen penetration through dense metal membranes based on Pd, according to the existing model, includes five main stages (Figure 11):

- Dissociative adsorption on the surface;
- Transfer of atomic hydrogen from the surface to the volume of Pd;
- Diffusion of atoms in the volume of Pd;
- Transfer from the volume of Pd to the surface;
- Recombinant desorption from the surface.

To describe this process, we will use the following notation:

*L* is the membrane thickness;

*C*_0_ is the relative volume concentration of H in the Pd array adjacent to the input surface;

*C_L_* is the relative volume concentration of H in the Pd array adjacent to the output surface;

*θ*_0_ and *θ_L_* are the degrees of saturation of the input and output surfaces of the membrane;

*N_V_* is the volume (in the membrane array) concentration of Pd;

*N_S_* is the surface (on the membrane surface) concentration of Pd;

*σ*_0_ and *σ_L_* are the roughness coefficients of the input and output surfaces;

*P*_0_ and *P_L_* are the external pressures of gaseous hydrogen at the input and output.

Considering the steady-state process of hydrogen penetration through the membrane, we will write out the hydrogen flows for each stage of this process. In this case, for the flows describing the transition of hydrogen through the surface and back, in contrast to [73], we will take into account the surface roughness.

The flow on the input surface of the membrane has the following form:

(2) $$J_{0}^{a-d}=J_{ads}(\sigma_{0}{,\theta }_{0},P_{0})-J_{des}(\sigma_{0},\theta_{0}),$$

The flow on the outlet surface of the membrane has the following form:

(3) $$J_{L}^{d-a}=J_{des}\left(\sigma_{L}{,\theta }_{L}\right)-J_{ads}\left(\sigma_{L},\theta_{L},P_{L}\right).$$

Here,$\;J_{ads}\left(\sigma ,\theta ,P\right)=2\sigma S\left(\theta \right)Q(P)$ is the adsorption flow, and $J_{des}\left(\sigma ,\theta \right)=\sigma k_{des}N_{S}N_{AA}(\theta )$ is the desorption flow.

The flow between the inlet surface of the membrane and the volume is as follows:

(4) $$J_{0}^{SV-VS}=J_{SV}(\sigma_{0}{,\theta }_{0},C_{0})-J_{VS}(\sigma_{0},\theta_{0,}C_{0}),$$

The flow between the volume and the outlet surface of the membrane is as follows:

(5) $$J_{0}^{VS-SV}=J_{VS}\left(\sigma_{L}{,\theta }_{L},C_{L}\right)-J_{SV}\left(\sigma_{L},\theta_{L,}C_{L}\right).$$

Here,$\;J_{sv}\left(\sigma ,\theta ,C\right)=\sigma k_{sv}(\theta )N_{s}N_{v}\theta (1-C)$ is the flow from the surface to the bulk of the metal, and $\;J_{VS}\left(\sigma ,\theta ,C\right)=\sigma k_{VS}(\theta )N_{S}N_{V}(1-\theta )C$ is the flow from the bulk of the metal to the surface.

The diffusion flow in the membrane has the following form:

(6) $$J_{diff}=-DN_{V}\frac{C_{L}-C_{0}}{L}.$$

#### 3.3.1. Formulation of the Stationary Problem and the Solution Technique

By virtue of the conservation law, the following equalities must be satisfied for the steady-state mode of hydrogen penetration through the membrane:

(7) $$J_{0}^{a-d}=J_{0}^{SV-VS}=J_{diff}=J_{L}^{VS-SV}=J_{L}^{d-a}=J.$$

Having solved the system of Equation (7), we can find the value of the hydrogen flux through the Pd membrane and the values of *θ*_0_, *C*_0_, *θ_L_*, and *C_L_* for which these equations are satisfied.

The system of Equation (7) was solved numerically using the iterative method; an accuracy of ε = 10^{−10}^ was considered achieved if the values of all flows differed by <ε.

#### 3.3.2. Dependencies and Parameters of the Model Used in the Calculations

The following dependencies are adopted to describe the process of hydrogen penetration through the membrane:

Adsorption modeling:

$$S\left(\theta \right)=\frac{S_{0}}{1+k_{ads}\left(\frac{1}{\theta_{00}}-1\right)},$$

$$\theta_{00}=1-\theta -\theta f(\theta ),$$

$$f\left(\theta \right)=\frac{2(1-\theta )}{1+\sqrt{1-4\theta \left(1-\theta \right)(1-e^{-\frac{E_{H}}{RT}})}},$$

$$Q(P)=C_{S}\left(P\right)\sqrt{\frac{RT}{2\pi M_{H_{2}}}}=\frac{P}{\sqrt{2\pi M_{H_{2}}RT}}.$$

Here, $C_{S}P=\frac{P}{RT}$ is the concentration of the gas phase adjacent to the surface.

Desorption modeling:

$$k_{des}=k_{0,des}e^{-2\frac{E_{des}}{RT}}.$$

$$N_{AA}=\frac{zN_{S}\theta }{2}F\left(\theta \right),$$

$$F\left(\theta \right)=1-f\left(\theta \right),$$

$$f\left(\theta \right)=\frac{2(1-\theta )}{1+\sqrt{1-4\theta \left(1-\theta \right)(1-e^{-\frac{E_{H}}{RT}})}},$$

Modeling the transition of H from surface to volume:

$$k_{SV}(\theta )=k_{0,SV}e^{-2\frac{E_{SV}}{RT}},$$

$$k_{0,SV}\left(\theta \right)=k_{0,VS}\frac{\sqrt[{4}]{T}}{10.154}\sqrt{\frac{F(\theta ){\left(1-\theta \right)}^{2}}{G(\theta )}},$$

$$F\left(\theta \right)=1-\left(\theta \right),$$

$$G\left(\theta \right)=\frac{1}{1+k_{ads}(\frac{1}{\theta_{00}}-1)},$$

$$\theta_{00}=1-\theta -\theta f\left(\theta \right),$$

$$f\left(\theta \right)=\frac{2(1-\theta )}{1+\sqrt{1-4\theta \left(1-\theta \right)(1-e^{-\frac{E_{H}}{RT}})}}\;.$$

Modeling the transition of H from volume to surface:

$$k_{VS}=k_{0,VS}e^{-2\frac{E_{VS}}{RT}}.$$

Modeling of flow in a membrane:

$$D=D_{0}e^{-2\frac{E_{diff}}{RT}}.$$

The model parameters and their values are presented in Table 2.

## 4. Conclusions

In this work, membrane materials based on Pd-Ag and Pd-Cu alloys were obtained that were capable of selectively passing hydrogen in a wide temperature range (298–773 K). The ability of Pd-based membranes to permeate hydrogen at low temperatures was achieved by activating their surface with a nanostructured coating. The study of the temperature corridor of efficiency showed the high performance of surface-activated membranes throughout the entire temperature range. Surface-activated membranes demonstrated the highest values of hydrogen flux over the entire temperature range, reaching up to 49.4 mmol s^−1^ m^−2^ for Pd-Ag membranes and up to 32.9 mmol s^−1^ m^−2^ for Pd-Cu membranes. The greatest difference in fluxes was recorded in the low-temperature range (from 298 to 473 K), where the greatest effect of surface activation was expected, since hydrogen transport is limited by surface processes. Membranes modified with filiform nanoparticles demonstrated a hydrogen flux up to 12 times higher than that of membranes with a smooth surface. The effect of nanostructured coating morphology on membrane permeability was also studied. Flux values for membranes modified with filiform nanoparticles were up to 1.5 times higher than those for membranes modified with classical nanoparticles. A theoretical model of hydrogen transport through metal membranes at low temperatures was developed on the basis of the results obtained. Experimental data on permeate flows successfully confirmed the predicted values of the model. This model allows for predicting hydrogen flows in the entire temperature range much more accurately compared to other existing models. The selectivity and stability of the developed membranes over a long period of operation without a loss of efficiency were confirmed. The study of the effect of surface activation of Pd-based membranes on the intensification of hydrogen permeability showed the success of the developed method, which in turn opens up wide possibilities for creating low-temperature, highly efficient membrane hydrogen filters based on palladium and other devices based on them.

## Figures and Tables

**Table 1:** Permeability parameters of palladium-based membranes studied under low-temperature conditions.

Membrane	Substrate	Thickness, μm	Temperature, K	∆p, MPa	J, mol s^−1^ m^−2^	Source
Pd	YSZ–γ-Al_2_O_3_	5	423	0.4	0.205	[64]
Pd-23%Ag	–	100	373	0.1	0.049	[65]
Pd-20%Ag	Porous α-alumina	3.2	373	0.2	0.31	[66]
Pd-23%Ag	–	84	573	0.5	0.09	[67]
Pd-34%Cu	α-Al_2_O_3_–γ-Al_2_O_3_	4	373	0.35	0.06	[68]
Pd-53%Cu	α-Al_2_O_3_–γ-Al_2_O_3_–ZrO_2_	3.5	373	0.5	0.06	[69]
Pd-30%Cu	Al_2_O_3_-PSS	23.74	448	0.1	0.007	[70]
Pd-23%Ag/Pd-40%Cu/nanothreads	–	30	373	0.1	0.07/0.09	This work
Pd-23%Ag/Pd-40%Cu/nanoparticles	–	30	373	0.1	0.05/0.06	This work
Pd-23%Ag/ Pd-40%Cu	–	30	373	0.1	0.02/0.01	This work

**Table 2:** Parameters and values used in the improved model.

Designation	Decoding	Meaning	Unit of Measurement
*N_S_*	Surface concentration of palladium	2.7078 × 10^−5^	mol m^−2^
*N_V_*	Bulk atomic concentration of palladium	1.09 × 10^5^	mol m^−3^
*R*	Universal gas constant	8.314462618	J mol^−1^ K^−1^
*L*	Membrane thickness	1.0 × 10^−6^	m
*S*_0_	Sticking coefficient at zero coverage	1.0	–
*k_ads_*	Coefficient associated with the probability of adsorption	0.05	–
*E_H_*	Pair interaction energy of hydrogen	2093.4	J mol^−1^
$M_{H_{2}}$	Molecular mass of hydrogen	0.002	kg mol^−1^
*P*_0_	H_2_ pressure on the left surface of the membrane	100,000	Pa
*P_L_*	H_2_ pressure on the right surface of the membrane	0	Pa
*k*_0_ *,_des_*	Desorption rate coefficient	4.8 × 10^17^	m^2^ mol^−1^ s^−1^
*E_des_*	Activation energy of desorption of atomic hydrogen	50,241.6	J mol^−1^
*z*	Number of nearest neighbors on the surface	4	–
*E_SV_*	Activation energy of transition from surface to bulk	55,684.44	J mol^−1^
*k*_0_ *,_VS_*	Coefficient of transition rate from bulk to surface	6.8 × 10^7^	m^3^ mol^−1^ s^−1^
*E_VS_*	Activation energy of transition from bulk to surface	22,190.04	J mol^−1^
*D*_0_	Diffusion coefficient	2.9 × 10^−7^	m^2^ s^−1^
*E_diff_*	Diffusion activation energy	22,190.04	J mol^−1^

## Data Availability

The data presented in this study are available on request from the corresponding author.

## Supplementary Materials

The supporting information can be downloaded at: https://www.mdpi.com/article/10.3390/ijms252312564/s1.
