# Supplementary material for: New Approaches to the Creation of Highly Efficient Pd-Ag and Pd-Cu Membranes and Modeling of Their Hydrogen Permeability
Source: Int J Mol Sci. 2024 Nov 22;25(23):12564. doi: 10.3390/ijms252312564 (PMC11641580; doi:10.3390/ijms252312564)
Supplement: Supplementary file 1 [file ijms-25-12564-s001.zip › Figure S1.pdf]

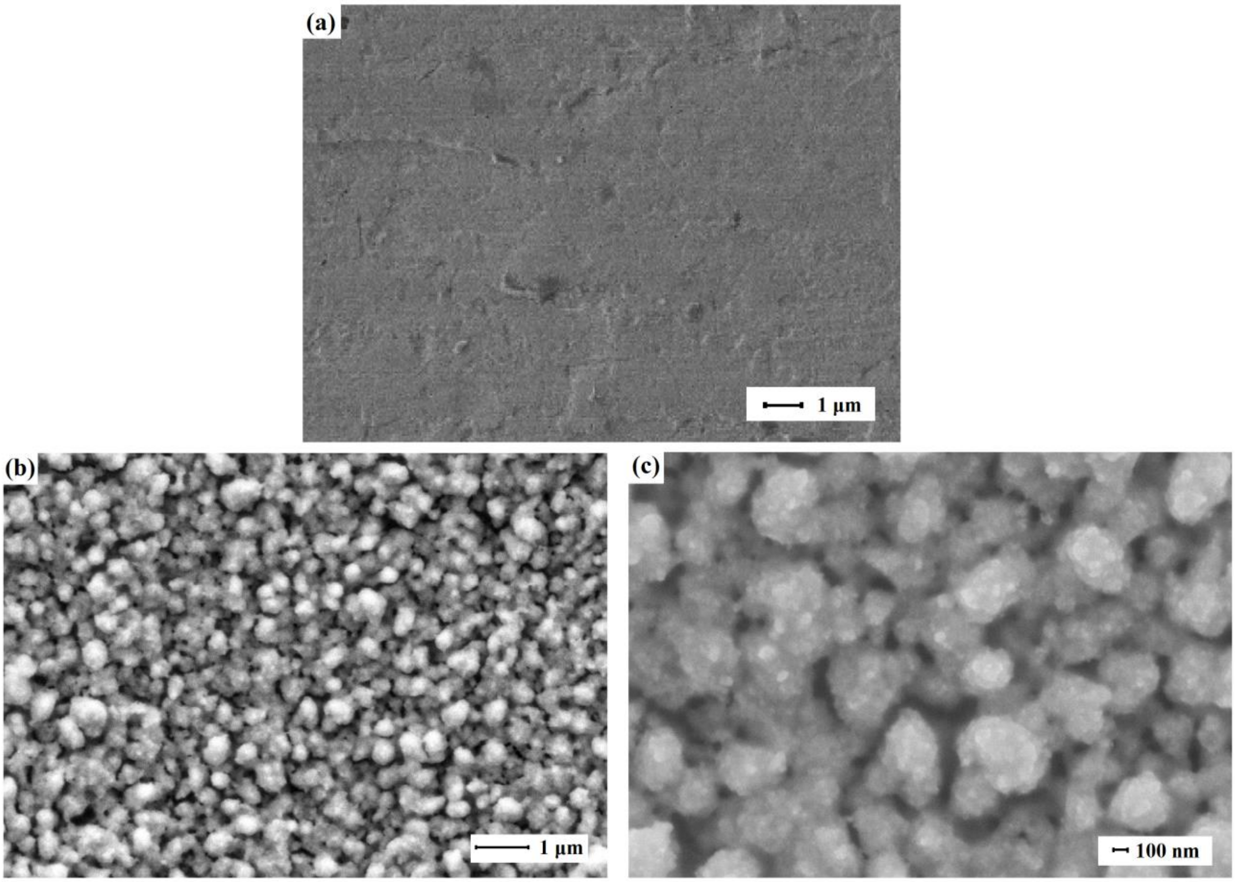

Figure S1. (a) SEM image of the surface of the unmodified Pd-Ag film. (b, c) SEM images of the modifying nanostructured palladium coating synthesized by the classical electrodeposition technique from a H_2PdCl_4 solution at a current density of 5-6 mA cm^{-2} and a time of 1.5-3 minutes.
