# Supplementary material for: New Approaches to the Creation of Highly Efficient Pd-Ag and Pd-Cu Membranes and Modeling of Their Hydrogen Permeability
Source: Int J Mol Sci. 2024 Nov 22;25(23):12564. doi: 10.3390/ijms252312564 (PMC11641580; doi:10.3390/ijms252312564)
Supplement: Supplementary file 1 [file ijms-25-12564-s001.zip › Figure S2.pdf]

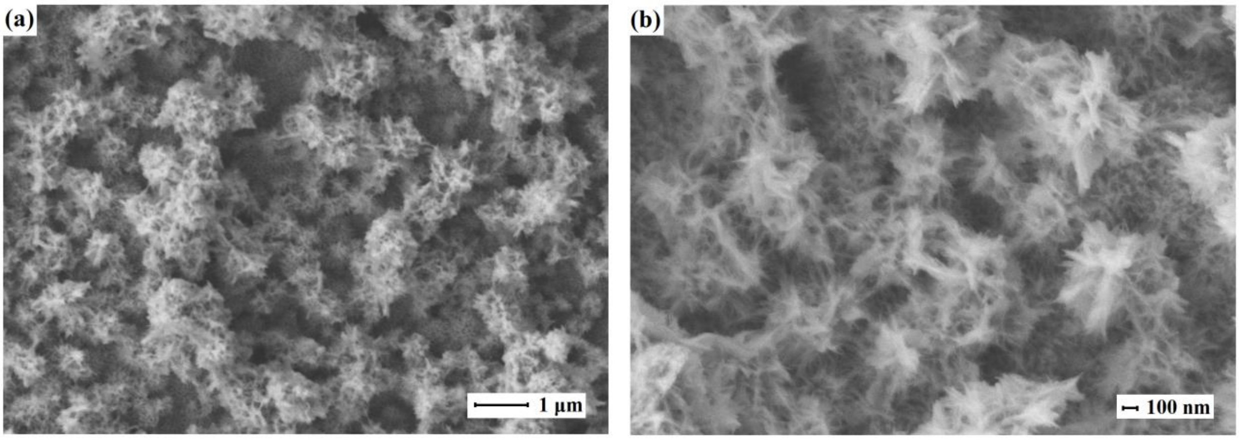

Figure S2. (a, b) SEM images of the modifying nanostructured palladium coating based on nanothreads synthesized by two-step electrodeposition from a solution of H_2PdCl_4 with $C_{19}H_{42}BrN$ at a current density of 2.5 μA cm^{-2} for 30–40 s and a current density of 0.35 mA cm^{-2} for 3–5 min.
